# Application of exosomes in tumor immunity: recent progresses

**DOI:** 10.3389/fcell.2024.1372847

**Published:** 2024-04-03

**Authors:** Haiyan Qiu, Junting Liang, Guang Yang, Zhenyu Xie, Zhenpeng Wang, Liyan Wang, Jingying Zhang, Himansu Sekhar Nanda, Hui Zhou, Yong Huang, Xinsheng Peng, Chengyu Lu, Huizhi Chen, Yubin Zhou

**Affiliations:** ^1^ School of Pharmacy, Guangdong Medical University, Dongguan, China; ^2^ The First Dongguan Affiliated Hospital, Guangdong Medical University, Dongguan, China; ^3^ Biomedical Engineering and Technology Lab, Discipline of Mechanical Engineering, PDPM Indian Institute of Information Technology Design and Manufacturing Jabalpur, Jabalpur, Madhya Pradesh, India

**Keywords:** extracellular vesicles, exosomes, tumor immunity, immunoregulation, drug delivery

## Abstract

Exosomes are small extracellular vesicles secreted by cells, ranging in size from 30 to 150 nm. They contain proteins, nucleic acids, lipids, and other bioactive molecules, which play a crucial role in intercellular communication and material transfer. In tumor immunity, exosomes present various functions while the following two are of great importance: regulating the immune response and serving as delivery carriers. This review starts with the introduction of the formation, compositions, functions, isolation, characterization, and applications of exosomes, and subsequently discusses the current status of exosomes in tumor immunotherapy, and the recent applications of exosome-based tumor immunity regulation and antitumor drug delivery. Finally, current challenge and future prospects are proposed and hope to demonstrate inspiration for targeted readers in the field.

## 1 Introduction

Cancer is a disease caused by a variety of factors that lead to abnormal cell proliferation and transformation, and which is a global disease that seriously threat human life and health (Sung et al., 2021). Currently, the main treatment for cancer includes traditional approaches such as chemotherapy, radiotherapy, and surgical treatment (Choudhury et al., 2019). Although these treatments can achieve rapid therapeutic effect, they may also lead to adverse reactions, poor patient compliance, tumor metastasis, and recurrence issues (Ferlay et al., 2019). Thus, finding an efficient and safe tumor treatment option is an urgent and significant problem. Emerging treatments such as tumor immunotherapy and immune-based advanced therapy have attracted great attention from researchers. At present, applied clinical immunotherapy includes Chimeric Antigen Receptor T-Cell Immunotherapy (CAT-T) drugs, cytotoxic T-lymphocyte antigen 4 (CTLA-4) inhibitors, and programmed cell death protein 1 (PD-1) or ligand 1 (PD-L1) inhibitors. Although tumor immunotherapy has good therapeutic effects, there are still bottlenecks, such as a low immune response rate due to reduce infiltration and functional exhaustion of immune cells, as well as immunosuppression in the tumor microenvironment (TME) (Rui et al., 2023). Thus, it is still urgent to develop new strategies to enhance the efficacy in immunotherapy.

**FIGURE 1:**
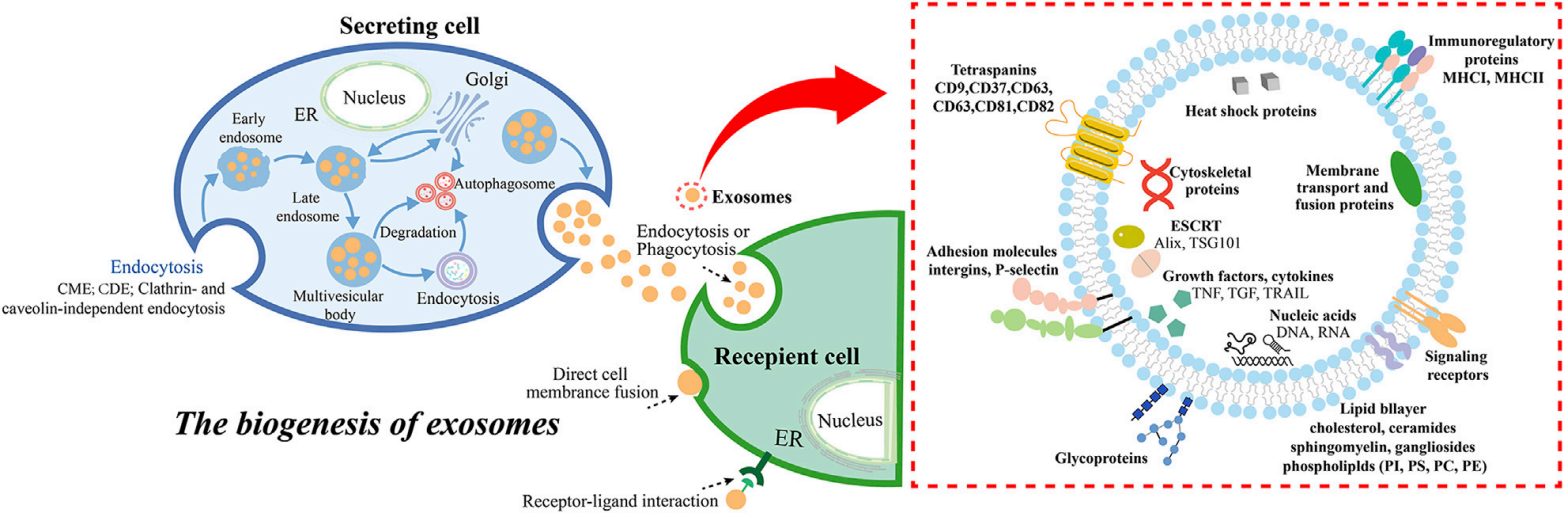
The biogenesis and secretion of exosomes, and the molecular compositions of the exosomes. Exosome biogenesis includes endosome formation CME, CDE, clathrin- and caveolin-independent endocytosis (McMahon and Boucrot, 2011), MVBs formation, cargo sorting, and extracellular release, while the ways in which exosomes interact with receptor cells generally include endocytosis (phagocytosis, micropinocytosis, lipid raft- or clathrin- or caveolin-mediated endocytosis), direct cell membrane fusion, and receptor-ligand interaction (He et al., 2018). Abbreviation: clathrin-mediated endocytosis (CME), caveolin-dependent endocytosis (CDE), endoplasmic reticulum (ER).

Exosomes have attracted broad attentions owing to their versatile roles in tumor immunity. Exosomes can influence nearby and distant cells to induce systemic immune responses and have been widely used in the occurrence and treatment of various tumors like breast cancer (Moradi-Chaleshtori et al., 2021), gastric cancer (Shen et al., 2022), and lung cancer (Wang et al., 2020). Exosomes themselves have certain advantages, such as immunogenicity, biocompatibility, and homing ability. Therefore, they are often used as immune regulatory molecules and drug delivery carriers (Kalluri and LeBleu, 2020). On the one hand, endogenous exosomes can affect the TME and promote tumor progression. On the other hand, it can affect the immune system and mediate immune activation or immunosuppression. For example, tumor-derived exosomes can affect stromal cells in the TME, induce macrophage polarization (Pritchard et al., 2020), promote T cell apoptosis and suppress proliferation (Dou et al., 2020), inhibit CD8^+^ T cell function (Chen et al., 2021), promote tumor growth, and induce immunosuppression. At the same time, exosomes can also express molecules that mediate immunosuppression, such as miR-183-5p, which upregulates PD-L1 expression (Luo et al., 2022), and lncARSR, which promotes the secretion of transforming growth factor β1 (TGF β1) (Li et al., 2022). Melanoma exosomes carrying the immune checkpoint PD-L1 can be up-regulated by IFN-γ to inhibit the tumor killing effect of CD8^+^ T cells and promote tumor growth (Chen et al., 2018). In addition, exosomal circGSE1 secreted by hepatocellular carcinoma cells (HCC) can induce the expansion of Treg cells by regulating the miR-324-5p/TGFBR1/Smad3 pathway, which promotes the secretion of immunosuppressive factors, inhibits the function of CD8^+^ T cells, and causes tumor immune escape (Huang et al., 2022). However, more researchers have focused on the antitumor studies of exosomes. Many studies have shown that tumor cells and dendritic cells (DCs)-derived exosomes carry a large number of tumor-associated markers, such as major histocompatibility complex class I molecules (MHC I) and heat shock proteins (HSP, HSP70, HSP50), which serve as essential signals for T cell activation (Fan et al., 2022), and producing antitumor immune responses in CD4^+^ T cells and CD8^+^ T cells. Additionally, exosomes can act as carriers for delivering chemotherapy drugs, photothermal agents, genes, protein, and other substances to achieve the purpose of treating tumors through synergistic immunotherapy. The application of exosomes in tumor immunity as regulatory molecules of antitumor immune responses and delivery carriers for antitumor drugs is worth exploring. This article reviews the formation, composition, function (Figure 1), isolation, characterization, and application of exosomes. It describes the current status of tumor immunotherapy while focusing on exploring exosome-based regulation of tumor immunity and drug delivery carriers to provide new insights into future tumor treatment.

## 2 Overview of exosomes

Exosomes are extracellular nanovesicles secreted by living cells such as tumor cells and immune cells under physiological and pathological conditions (Bang and Thum, 2012). They are widely presented in various body fluids. Exosomes play an important role in intercellular communication and material. Some exosomes retain information from donor cells and are often used as carriers for substance and information exchange.

### 2.1 Exosomes biogenesis

According to the origin, formation, and size of extracellular vesicles (EVs), which are classified into exosomes (30 nm–150 nm), microvesicles (100 nm-1 μm), and apoptotic bodies (50 nm-5 μm) (Kalluri and LeBleu, 2020). Exosomes are generated by cell membrane to form several small vesicles, which fuse with each other to form early endosomes (Hessvik and Llorente, 2018), and then interact with the Golgi apparatus to form late endosomes, which further form multivesicular bodies (MVBs) containing intraluminal vesicles (ILVs) (Yue et al., 2020; Rahmati et al., 2023b). Meanwhile, cargos in exosomes are sorted into MBVs, including proteins, nucleic acids, and lipids. The cytoplasmic components are engulfed and enclosed within ILVs, and finally, a portion of the MVBs is degraded by lysosomes, while another portion is involved in the development of special organelles like melanosomes (Tkach and Théry, 2016; McAndrews and Kalluri, 2019). The other part is through SNARE complexes by such as v-SNAREs (on vesicles), t-SNAREs (on target membranes), Rab GTPases, tethers, and additional proteins fused with the cell membrane, for example, one V-SNARE and two T-SNARE form a four-helix bundle, which promotes MVB fusion with the cell membrane (Krylova and Feng, 2023), and finally releases ILVs into the extracellular through exocytosis, budding and intracellular plasma membrane-connected compartment (IPMC) contraction (Yue et al., 2020; Chen et al., 2021c). The released exosomes are involved in material transport and signal transduction through endocytosis, cell membrane fusion and surface receptor binding (Yu et al., 2024).

Studies have shown that the formation of ILVs is closely associated with the endosomal sorting complex required for transport (ESCRT) present in the endosomal system (Kowal et al., 2014; Krylova and Feng, 2023). The ESCRT is a complex protein machine whose main function is to promote the degradation of membrane proteins labeled by ubiquitin, and is also related to cell division and budding (Juan and Fürthauer, 2018). The ESCRT system consists of five independent protein complexes (ESCRT-0 to ESCRT-III, Vps4), which participates in the isolation and classification of membrane proteins, causing the endosomal boundary membrane to indent inward to form MVBs, and the Vps4 complex transmits the load to the vesicle, synergistically promotes vesicle budding. Studies have reported that the typical exosome protein Alix (Baietti et al., 2012; Roucourt et al., 2015) associates with several ESCRT proteins (TSG101 (Palicharla and Maddika, 2015) and CHMP4 (Colombo et al., 2013; Marie et al., 2023). Additionally, ESCRT-0 and ESCRT-I proteins are related to budding. Depletion of these proteins reduces exosome production, while cleavage of the ESCRT-III protein at the bud neck promotes vesicle isolation, while its knockdown induces exosome secretion. These results suggest that the ESCRT machinery is essential for exosome biogenesis. Other studies have found that exosomes formation occurs independently of ESCRT but requires sphingolipid ceramide instead. The purified exosomes are found to be rich in ceramide, and the formation of ceramide are reduced by neutral sphingomyelinase inhibition, leading to decreased budding of the MVBs membrane and the release of exosomes. This indicates that ceramide forms exosomes in an ESCRT-independent pathway (Trajkovic et al., 2008b; Wei et al., 2020). In addition, we do not discuss the mechanisms of exosomal internalization in detail here, and the reader can refer to a recent review of the topic (Arya et al., 2024).

### 2.2 General compositions of exosomes

Exosomes contain proteins that are related to the formation of the endosomal pathway (Keerthikumar et al., 2016). They not only contain selective assembly proteins of protein components in the donor cells, known as cell-specific proteins, which include major histocompatibility complexes (MHC I, MHC II) and Fas ligand (Fas L), facilitating exosome source recognition, but also contain marker proteins commonly found in exosomes, including membrane transport essential intraluminal vesicle sorting complexes (Alix) and tumor susceptibility gene 101 (TSG101), tetraspanin superfamily proteins (CD9, CD63, CD81), HSP70, HSP90, integrins, and so on (Théry et al., 2009). Additionally, exosomes also contain nucleic acid materials such as double-stranded DNA (dsDNA), messenger RNA (mRNA), microRNA (miRNA), long non-coding RNA (lncRNA), and circular RNA (circRNA) (Boriachek et al., 2018). Numerous studies have shown that exosomes from different cell sources exhibit diverse nucleic acids and proteins, which are related to cell types and physiological or pathological conditions.

In general, the sorting mechanism of exosomes from normal cells and tumor cells mainly include ESCRT-dependent and ESCRT-independent pathways, but the specific sorting mechanisms are still different. Sorting of exosomal protein cargo includes the sorting of ubiquitin (a post-translational modification that makes it a target for proteasomal degradation, or re-transported back to the ER or Golgi) proteins into exosomes through the precise labor allocation of ESCRT complexes, such as Alix, HD-PTP, CHMP4, and Vps4 (Chen et al., 2021c). In addition, Sorting mechanisms include small ubiquitin-like modifiers (SUMO) (Geiss-Friedlander and Melchior, 2007) and Ubiquitin-like 3 (UBL3) (Welchman et al., 2005) such as PTMs, the lipid raft (stomatin and flottlin-1) (Skotland et al., 2019; Dawson, 2021) and ESCRT-independent pathway (Trajkovic et al., 2008a). For example, the Alix-dependent pathway of ESCRT-dependent pathway recruits ESCRT-III to MBVs by the Syndecan-Syntenin-Alix pathway (Majer et al., 2019). ESCRT-independent pathways include The nSMase2-ceramide-dependent pathway, Caveolin-1, Flotillins, Cholesterol and so on (Han et al., 2022). For the sorting of nucleic acid cargo in exosomes, RNA is involved in RNA binding protein (RBP) with major sequence specific RNA binding domain, and miRNA is involved in Ago2 and FMR1 (Teng et al., 2017; Groot and Lee, 2020), lncRNA involves hnRNPA2B1 and hnRNPA1 (Chen et al., 2019; Chen et al., 2021), circRNA involves SNF8 and hnRNPA2B1 (Pan et al., 2022). Deoxynucleotides are mediated by ESCRT-independent mechanisms and interacting with transmembrane proteins (Jeppesen et al., 2019). There are still slight differences in the mechanism of exosomes derived from normal cells and tumor cells. For example, hnRNPA2B1 negatively regulates miR-503 sorting to endothelial cell-derived exosomes (Pérez-Boza et al., 2020). Synaptophysin binding cytoplasmic RNA interacting protein (SYNCRIP) binding motif can regulate the localization of hepatocyte exosomal miRNA through GGCU sequence (Santangelo et al., 2016). Knocking down Vault protein (MVP) can increase the expression of miR-193a through immunoprecipitation, while the expression of miR-193a in colon cancer cell exosomes is opposite (Teng et al., 2017). Arginine 2(Ago2) can bind to exosome mirnas to form AGO2-mirna complex and regulate the sorting of miRNAs in exosomes. KRAS oncogene can promote phosphorylation of S387 and inhibit the sorting of Ago2-miRNA complex to exosomes (McKenzie et al., 2016). Neutral sphingomipinase 2 (nSMase2) regulates the release of miR-210 in metastatic cancer cell (Lallemand et al., 2018). Micronuclei (MN) of cancer cells are released when the nucleus is unstable to damage the nuclear membrane and release gDNA-containing exosomes (Fenech, 2020).

### 2.3 Isolation and characterization of exosomes

**FIGURE 2:**
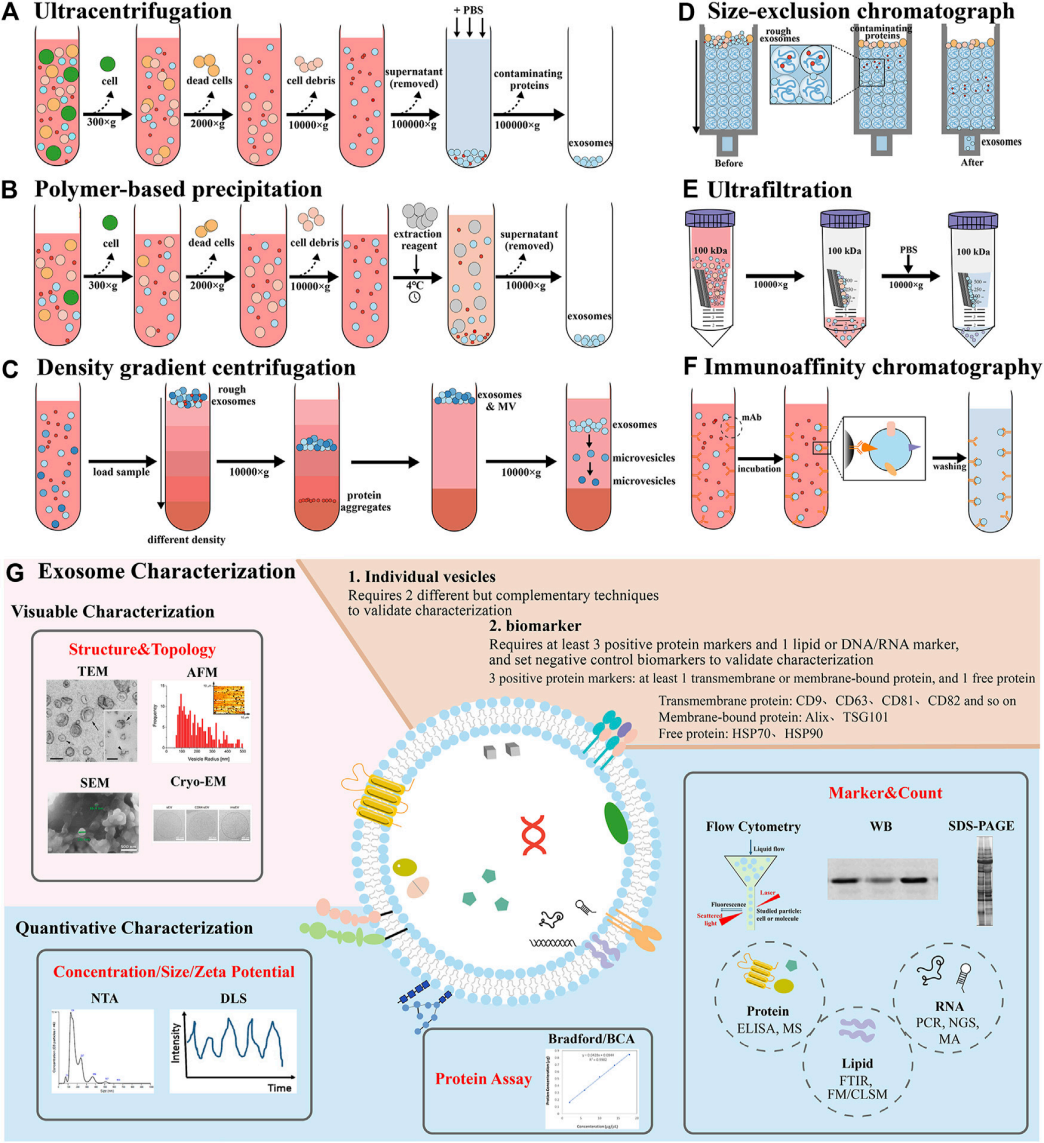
Demonstration of various isolation and characterization methods of exosomes. Isolation methods include **(A)** ultracentrifugation, **(B)** polymer-based precipitation, **(C)** density gradient centrifugation, **(D)** size-exclusion chromatograph, **(E)** ultrafiltration, **(F)** immunoaffinity chromatography. **(G)** Exosome characterization techniques contain visual characterization and quantitative characterization, including structure and topology, concentration, size and zeta potential, protein assay, biomarker and count (Théry et al., 2006; Szatanek et al., 2017; Santucci et al., 2019; Al Mannai et al., 2022; Dong et al., 2023). Abbreviation: enzyme linked immunosorbent assay (ELISA), mass spectrometry (MS), fourier transform infrared spectroscopy (FTIR), fluorescence microscopy (FM), confocal laser scanning microscope (CLSM), polymerase chain reaction (PCR), next-generation sequencing (NGS), microarray analysis (MA).

The isolation of exosomes is mainly based on their size, density, solubility, charge, and immune affinity properties. General methods Include ultracentrifugation (UC) and density gradient centrifugation, polymer precipitation (commercial kit), size exclusion chromatography (SEC), ultrafiltration, and immunoaffinity chromatography (Yang et al., 2020) (Figure 2). Ultracentrifugation is a classical method for exosome extraction based on the density and size of exosomes, including differential centrifugation (Staubach et al., 2021). And it can be used for large volume samples. However, its purity is limited, the instrument is expensive, and it is often combined with other methods. For example, the samples obtained by ultracentrifugation are subjected to density gradient centrifugation to improve the purity of the isolated exosomes, and the media of density gradient centrifugation included iodixanol, sucrose and so on (Nikfarjam et al., 2020). The polymer precipitation method (commercial kit) is based on the polymer reduces the solubility of exosomes to form precipitation to separate the exosomes (Atha and Ingham, 1981; Kurian et al., 2021), and the main medium is polyethylene glycol (PEG). Its yield is high but its purity is low. These separation methods are difficult to separate exosomes from apoptotic bodies and microcapsules, and the structural integrity and biological activity of exosomes will be damaged due to the shear stress or changes in the medium. Size exclusion chromatography is based on size separation, multiple SEC columns can separate different extracellular vesicle subsets and can be used to separate other protein impurities in exosomes, with high purity and more uniform size, and protect the biological function of exosomes (Monguió-Tortajada et al., 2019; Guo et al., 2021). Ultrafiltration is mainly used to separate the vesicles and exosomes of different sizes according to the retained molecular weight. Its purity is high, and there is no significant change in the integrity of exosomes, but there is membrane loss, protein pollution and structural changes. However, tangential flow filtration (TFF) can pass the liquid of different subsets of extracellular vesicles through the membrane, and the small ones pass through the membrane, while the large ones are retained on the membrane to circulate and concentrate (McNamara et al., 2018), which can be used for large-scale separation. For example, hydrostatic filtration dialysis (HFD) uses filter-concentration-dialysis to separate different subgroups with low damage (Musante et al., 2014). Immunoaffinity chromatography is based on the specific membrane proteins of exosomes (such as CD81, CD63, CD9, Alix, EpCAM) to the corresponding antibodies or ligands on immobilization media (magnetic beads or polymeric materials, e.g., agarose beads and monolithic columns) (Sharma et al., 2018; Wijerathne et al., 2020), and can be used to separate different exosomes subpopulations, but it is not suitable for large samples. With the exploration of exosome isolation technology, some new technologies have been found, such as microfluidic technology deterministic lateral displacement (Liangsupree et al., 2021), which uses the size, density and viscoelasticity of exosomes to achieve different exosome subpopulation isolation through immune affinity or physical field, but the yield is limited (Yang et al., 2019; Wu et al., 2022). Another example is the application of Flow field-flow fractionation (FFF) in exosome isolation, which uses size for separation, such as asymmetric flow field-flow fractionation (AsFlFFF/AF4), which has been used to separate different subpopulations (such as exopolymers and exosomes) (Sitar et al., 2015). EXODUS ^®^ is a novel exosome isolation technology based on negative pressure oscillation and dual coupled ultrasonic oscillation (Chen et al., 2021d). Another example is DNA nanotechnology, where DNA aptamer sequences (such as CD63 aptamer) are used as hydrogels for specific encapsulation through affinity interactions, and released by enzymatic degradation and strand displacement, so as to achieve selective and non-destructive separation of exosome subpopulations (Tang et al., 2023). In addition, Surface-enhanced Raman scattering (SERS) and Single-molecule array (Simoa), based on SARS-CoV-2 virus, can be used for the isolation of exosomes and other subpopulations (Ma et al., 2024). And the International Association for the study of extracellular vesicles (ISEV) pointed out in the “2018 guidelines for minimum Information of extracellular vesicles” (Théry et al., 2018) that there is no single separation method at present, and multiple methods are often used to enrich exosomes. Therefore, it is necessary to continue to explore the isolation methods of exosomes which are purer, faster, more reliable, and capable of mass production.

The isolated exosomes need to be characterized to evaluate the effect, biomarker or function and purity of the separation (Lai et al., 2022). ISEV pointed out in the “2018 minimum Information Guide for extracellular vesicle Research” that more than three positive protein markers should be detected (Théry et al., 2018), and at least one transmembrane protein (CD9, CD63, CD81, CD82, and so on.) or membrane binding protein (Alix, TSG101), one cytoplasmic protein (HSP70) should be detected, and a negative control biomarker should be set up and characterized mainly by Western blotting (WB) and flow cytometry (FCM) (Foster et al., 2016). The detection of individual vesicles requires two different and complementary methods, such as scanning electron microscope (SEM), transmission electron microscope (TEM), cryogenic electron microscope (Cryo-EM) and atomic force microscope (AFM) to observe the shape and size of the exosomes, and nanoparticles tracer analysis (NTA) and dynamic light scattering (DLS) to detect the concentration, particle size and Zeta potential of the exosomes (Tiwari et al., 2021). The concentration of total protein is often determined by BCA.

## 3 Overview of exosome functions and applications

### 3.1 Exosomes functions

Exosomes have been suggested to function as cellular garbage bags in previous studies, expelling excess or unnecessary non-functional cellular components and maintaining the stability of the intracellular environment. However, exosomes are produced by multiple endocytic pathways, involving the selective combination of cell surface proteins, and signaling molecules, and therefore carry specific biological substances related to their source cells, that is, the heterogeneity of exosomes, which can regulate and transmit signals from donor cells. Exosomes are an important intercellular communication and material transfer molecules, which carry information and contents of donor cells and transmit them to recipient cells, simulating the interaction between cells, with high biocompatibility, stability, natural homing ability, low toxicity, and can reduce clearance by the mononuclear phagocyte system (MPS). They are often used as natural endogenous transporters carriers for intercellular communication. It has been discovered that exosomes play important roles in many biological processes, including the presentation of antigen, angiogenesis, and intercellular signal transduction. On the one hand, exosomes can promote epithelial-mesenchymal transition (EMT) of tumor cells (Bhattacharya et al., 2023), induce angiogenesis (Jiang et al., 2022), mediate immune escape, regulate macrophage polarization and lipid metabolism to promote tumor development (Ye et al., 2023). For example, LncRNA BCRT1 can promote M2 polarization of macrophages, promote tumor migration and chemotactic ability, and thus promote tumor progression (Ye et al., 2023). On the other hand, different cells carry different antigens, which affect the anti-tumor functions of immune cells such as DC cells, T cells, macrophages and so on (Zhang and Yu, 2019). For example, tumor-associated macrophage (TAM) exosomes are secreted by Akt to promote Rab27a activation by MADD and increase PD-L1 expression, thereby inhibiting CD8^+^ T cells (Zhong et al., 2023a). MSCS derived exosomes can activate VEGF receptor and NF-κB pathways by increasing ICAM-1 expression, hypoxia inducible factor (HIF) -1α and Wnt4/β-catenin pathways, activate AKT signaling pathway, and target PTEN signaling pathway. It promotes angiogenesis in an angiostatin 1-dependent manner (Zhu et al., 2023). In addition, plant-derived exosomes can be loaded with antioxidants and promote the production of antioxidant molecules such as Bcl2, HO-1 and NQO-1 to reduce ROS expression and achieve antioxidant effect (Rahmati et al., 2023a). At the same time, plant-derived exosomes can activate adenosine monphosphate activated protein kinase (AMPK) and AhR/COPS8 pathway, inhibit the expression of TNF-α, IL-1β and IL-6 pro-inflammatory factors, promote the expression of IL-10 and IL-22 anti-inflammatory factors, and increase the tolerance of *Lactobacillus* to bile to prevent or alleviate inflammation (Yi et al., 2023). Consequently, they regulate physiological homeostasis and pathological processes associated with diseases like tumors and infections. For example, exosomal lncRNA derived from tumor is considered a signaling mediator that coordinates neighboring tumor cells functions (Wang et al., 2019). Exosomes released from pancreatic β cells transmit apoptotic signals by transferring microRNA to neighboring β cells (Guay et al., 2015). Exosomal miR-3153 derived from lung adenocarcinoma cells can induce M2 macrophage polarization by activating the JNK signaling pathway (Xu et al., 2023). Exosomes derived from adipose tissue macrophages obtained from obese donors can regulate insulin sensitivity both *in vivo* and *in vitro*; that is, exosomal miR-155 can be transferred to insulin target cells through paracrine or endocrine regulatory mechanisms to affect cellular insulin action, insulin sensitivity, and overall glucose homeostasis *in vivo* (Ying et al., 2017). In short, these findings suggest that exosomes can regulate intercellular communication through information molecules to modulate physiological and pathological processes, demonstrating the potential of carriers for delivery *in vivo*.

### 3.2 Applications of exosomes

Exosomes derived from different cells have different components and functions. On the one hand, they can be used for early diagnosis and monitoring of diseases; on the other hand, it can be used for disease treatment. In the application of detection, it can be used for central nervous system diseases, cardiovascular diseases, and tumors. For instance, exosomes derived from tumor cells contain specific miRNAs, which can be used as markers for early tumor diagnosis (Wang et al., 2022). In the field of treatment, it can be used in immune response and infection, metabolic and neovascular diseases, neurodegeneration, cancer and so on, showing great potential in the applications of drug delivery (Kalluri and LeBleu, 2020). The effect of exosomes on tumors is mainly manifested in antitumor immunity, immunosuppression, and tumor immune escape, in order to inhibit or promote the occurrence, development, invasion, and metastasis of tumor cells. Tumor-derived exosomes contain tumor antigens, which can activate antigen-presenting cells (APCs) and induce immune responses to enhance immune ability. Exosomes demonstrate high biocompatibility, high stability (Ha et al., 2016), low toxicity, low immunogenicity, natural homing ability, can cross the plasma membrane and blood-brain barrier and other biological barriers, and can be used as a carrier to deliver small molecular chemotherapeutic drugs, proteins, nucleic acids and genes. That can avoid MPS clearance, prolong the body circulation time and immune escape, reduce drug toxic side effects, protect drug activity and stability, and achieve targeted therapy of tumors (Zhuang et al., 2011). In addition, exosomes can also affect other biological processes. For example, DCs-derived exosomes can carry tumor antigens to lymph nodes and then transfer them between different DC subsets to stimulate specific immunity and regulate the host immune response, while exosomes derived from Treg cells can induce immune tolerance. Bone marrow MSCs contain a variety of miRNAs, which can promote new angiogenesis (Huang et al., 2015) and activate the Hedgehog signaling pathway to promote tumor growth (Shi et al., 2019).

## 4 Tumor immunity

Tumor immunotherapy is the fourth type of tumor treatment after traditional therapy and shows great therapeutic potential. It is a hot field of international research. However, the existing tumor immunotherapy has its own limitations and low clinical responsiveness, including insufficient activation of antitumor immune cells, limited tumor infiltration, and limited immune activity (Rui et al., 2023). The exosome is an endogenous nano-extracellular vesicle, that carries the specific components of donor cells, and plays an important role in mediating intercellular communication and tumor immunotherapy. In recent years, researchers have taken advantage of the good biocompatibility, homing, and low immunogenicity of exosomes to enhance antitumor immunotherapy. The following will describe tumor immunotherapy and current exosome-based tumor immunity.

### 4.1 Overview of tumor immunotherapy

**FIGURE 3:**
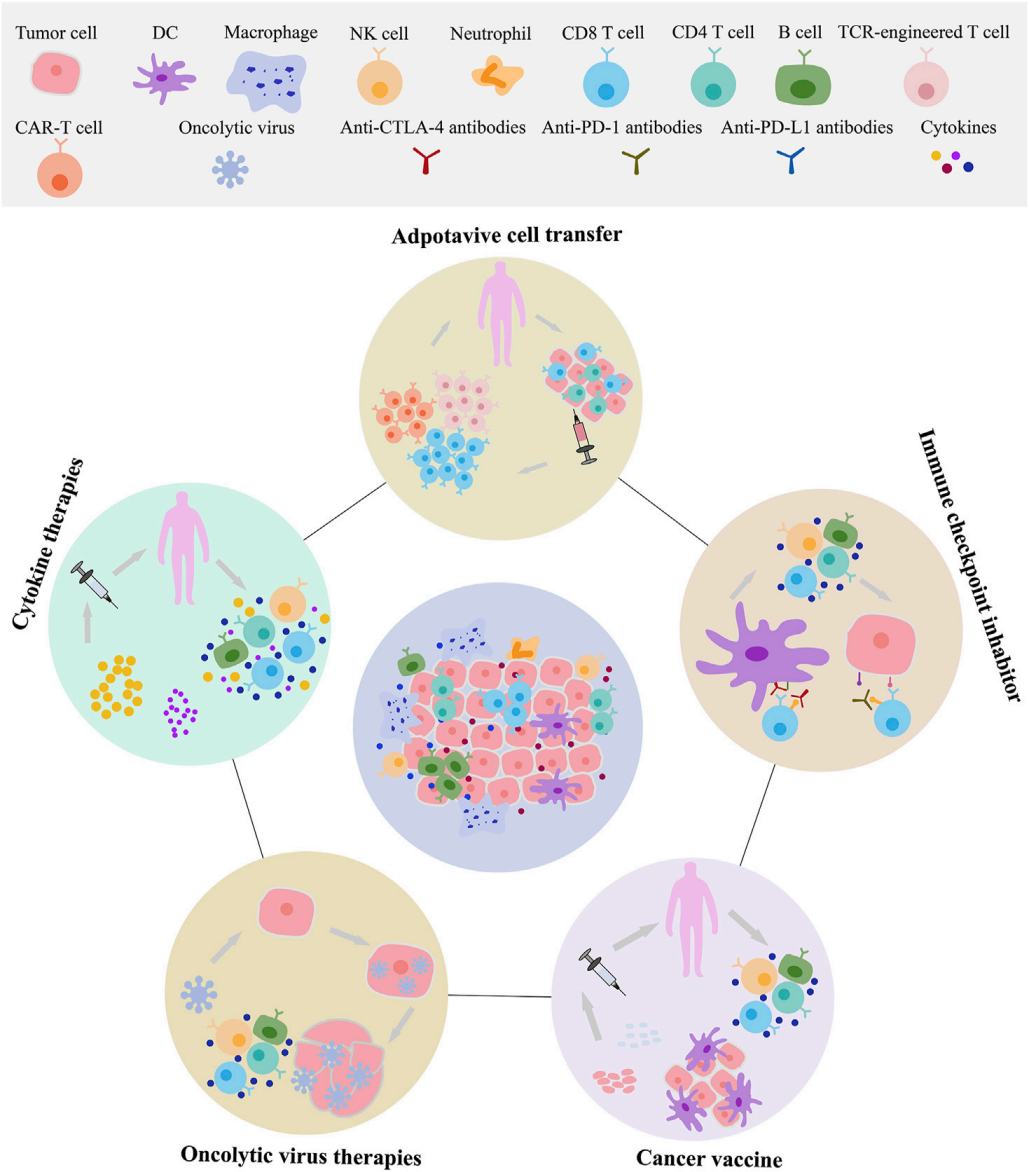
The main category and application of tumor immunotherapy. Exosomes can be involved in adoptive immune cell therapy, immune checkpoint, cancer vaccines, oncolytic virus therapies. These immunotherapies include T cells, natural killer cells (NK), dendritic cells (DCs), macrophages, and others.

Tumor immunotherapy is an emerging method of tumor therapy in recent years that mainly activate internal immune system of the patients, enhance the immune response of antitumor, and specifically clear the minimal residual tumor focus and killing tumor cells. At present, such treatment includes adoptive immune cell therapy (Chimeric antigen receptor T cell CAR-T, T cell receptor chimeric T cell TCR-T, DCs, Cytotoxic T lymphocyte CTL, Natural Killer cell NK, and so on), immune checkpoint inhibitors, cancer or neoantigen vaccines, oncolytic virus therapies, cytokine therapies, genetically engineered modified immune cell therapy, monoclonal antibody immunotherapy, neoantigen vaccines, and so on (Figure 3). Broadly, tumor immunotherapy can be divided into two categories: non-specific and tumor antigen specific. Non-specific methods include non-specific immune stimulation and immune checkpoint inhibition, while tumor antigen-specific approaches include tumor vaccines and adoptive immune cell therapy.

### 4.2 Exosome-based tumor immunotherapy

The exosomes secreted by cells are bidirectional in tumor immunity (Xu et al., 2020). On the one hand, it can activate immunity and inhibit tumor growth, proliferation, and metastasis, which are often used as an antitumor vaccine and targeted antigen/drug carrier. Exosomes derived from tumor cell carry tumor-associated antigens and several HSPs (such as HSP70 and HSP90), which can promote the effective antigen layer of APCs and stimulate the immune response to tumor cells. For example, adoptive immune cell CAR-T cells and exosomes can reduce the expression of Ki-67, Gzms-B, IFN-γ and TNF-α, and improve CD8^+^ T cell invasion and activation (Zhong et al., 2023b), and exosomes containing CAR express high levels of cytotoxic molecules that significantly inhibit tumor growth (Fu et al., 2019). Melanoma exosomes carrying the immune checkpoint PD-L1 can be upregulated by IFN-γ to inhibit the tumor killing effect of CD8^+^ T cells and promote tumor growth (Chen et al., 2018). Tumor-derived exosomes carry MHC-I and tumor antigens on their surface and induce T lymphocyte responses, which can be developed into tumor vaccines (Xu et al., 2020). Exosomes derived from tumor cells infected with OVS activate the immune system by increasing the levels of MHC and costimulatory molecules in DC (Labani-Motlagh et al., 2021). As found in earlier studies, antigens in the exosomes derived from lymphoma can be transferred to DCs, presenting and activating specific CTLs (Andre et al., 2002). For example, exosomes derived from B cells can directly stimulate T cell immune response (Raposo et al., 1996), and DC cell-derived exosomes can induce specific CTL responses (Zitvogel et al., 1998), so as to achieve the purpose of antitumor. There are also many studies using exosomes as drug delivery carriers to deliver antitumor drugs such as paclitaxel (PTX), doxorubicin (DOX), the RAB27a protein, the CD47 gene, and so on. On the other hand, exosomes can promote tumor metastasis, mediate tumor immunosuppression, allow immune escape, and increase immune tolerance. For example, low expression of MHC I and MHC II on the surface of tumor cells or expression of immunosuppressive molecules such as vascular endothelial growth factor (VEGF), interleukin-10 (IL-10), Fas ligand (Fas-L), TGF-β and prostaglandin E2 (PGE2) to mediate immune escape. It has also been reported that interleukin 8 (IL-8), interleukin 6 (IL-6), and VEGF expressed on the surface of exosomes, affect the Wnt signaling pathway (Menck et al., 2013) to mediate tumor invasion and metastasis.

## 5 Exosomes as tumor immunomodulatory molecules

**FIGURE 4:**
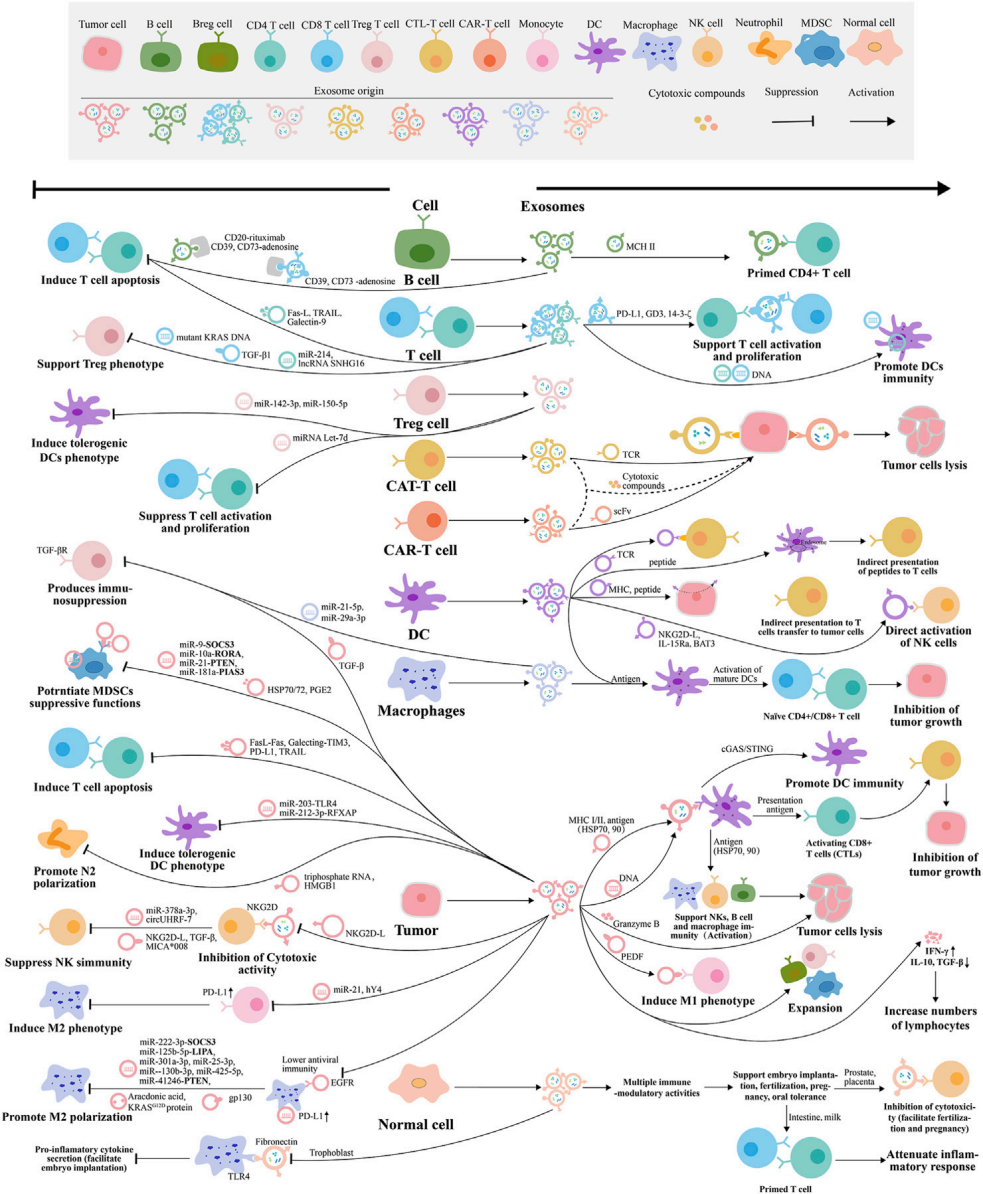
Tumor immunomodulatory mechanism of exosomes released by different cells. The exosomes are represented by the same color as the host cells.

Exosomes are small membrane vesicles produced by late endosomes that carry many signaling molecules involved in immune response and signal transduction. Exosomes participate in innate immune responses and adaptive immune responses by regulating immune responses and mediating antigen presentation (Figure 4). On the one hand, exosomes mediate the transport of antigens, proteins, cytokines, and other related substances between monocytes, macrophages, DCs, and NK, regulate tumor immunogenicity, activate, or suppress immune effector cells, promote immune response, and thus participate in innate immune regulation. On the other hand, exosomes mediate the uptake and processing of antigens by APCs such as DCs and B lymphocytes, the exchange of proteins, lipids and nucleic acids, and induce antigen presentation processes such as activated T lymphocytes, thus participating in adaptive immunity regulation (Rožman, 2018; Walker et al., 2019). Early reports have reported that EVs induce antitumor immune responses through innate and adaptive immunity (Buzas, 2022). For example, EVs derived from antigen-presenting cells transmit signals to regulate adaptive immune responses (Lindenbergh and Stoorvogel, 2018). Exosomes show different interactions in the immune system, such as the dual regulation of tumor immunity, which can not only activate immunity to achieve the purpose of antitumor, but also inhibit immunity and promote tumor growth. Under normal physiological conditions, exosomes can participate in antigen presentation and immune activation, while under pathological conditions such as tumors, they can cause immune escape and promote tumor growth. Source of immune cells and some tumor cells secrete exosomes can activate the immune cells, regulate lymphocyte and function of bone marrow cells, direct or indirect effects on tumor cells, or as a tumor vaccine. Exosomes derived from tumor cells can carry themselves immunosuppressive molecules and factors, which can promote immunosuppression, tolerance, inhibit the function of immune effector cells, or prevent immune activation, DCs maturation, NK and T cell-mediated cytotoxicity, affect the development, maturation, and antitumor effect of immune cells, and promote tumor escape. Therefore, the involvement of exosomes as tumor immune regulatory molecules in the progression of tumor immune activation and suppression (Table 1) is reviewed below.

**TABLE 1:** Immunomodulatory effects of exosomes.

Exosome origin		Isolation	Functional molecules	Tumor type	Effector cells	Effects	Refs
Normal cell or tissue	Adipocyte	Ultracentrifugation	miR-27a-3p	LUAD	ICOS^+^ T cells, CD4^+^ T cells	Promote ICOS^+^ T cells proliferation and IFN-γ secretion	Fan et al. (2020)
	—	Ultracentrifugation	aCD3-aEGFR-PD-1-OX40L	TNBC	CD8^+^ T cells, Treg cells	Increase CD8^+^ T cells infiltration and reduce Treg cells immunosuppression	Cheng et al. (2022b)
	hBMSC	Ultracentrifugation	miR-1913	OS	—	Target inhibition of NRSN2 expression, inhibit the tumor cell viability, proliferation, migration, and invasion	Zhou et al. (2021a)
	CAF	Exosome isolate kit	hsa-miR-139-5p, ACTR2 and EIF6	OSCC; Cal-27 cells	—	Dysregulate mRNA and miRNA between cells, mediate the CD81, PIGR, UACA, and PTTG1IP genes, induction of tumor resistance, invasion, and growth	Wang et al. (2023b)
	CAFS	Iodixanol density gradient, Ultracentrifugation	miR-92	BLCA	T cells; NK	Downregulation of LATS2, enhances the nuclear translocation of YAP1 and the transcriptional activity of PD-L1, and inhibits immune cells function	Dou et al. (2020)
	Tissue fluid	mini-SEC	CD45	HNSCC	CD8^+^ T cells, Treg cells	Exosomes carry adenosine, a suppressor factor that mediates immunosuppression, inducing CD8^+^ T cells apoptosis and Treg differentiation	Beccard et al. (2020)
	Tissue fluid	Ultracentrifugation	miR-3184-3p	GBM	M2 Macrophages	Inhibition of RSAD2, and directly promoted glioma progression, and M2-like macrophage polarization	Xu et al. (2022a)
Immune cell	DCs	Ultrafiltration	MHC I, MHC II	Melanoma; B16F10 cells	T cells	Activate T cells and act as a “bridge” between cancer cells	Fan et al. (2022)
	Macrophages	Ultracentrifugation	miRNA-16-5p	GC	T cells	Downregulate PD-L1, activate T cell immune response	Li et al. (2020)
	Macrophages	Ultracentrifugation	—	Tumor	Macrophages	Transformation into M1-type macrophages with high MHC II expression directly guides TAM reprogramming	Kim et al. (2023)
	Macrophages	Ultracentrifugation	microRNA-155-5p	CC	CD3^+^/IFN-g^+^ T cells	Transfer miR-155-5p to tumor cells and downregulate ZC3H12B and upregulate IL-6 to promote immune escape	Ma et al. (2021)
	Macrophages	Differential centrifugation	miRNA-23a-3p	OSCC; Cal-27 cells	—	Transfer miRNA-23a-3p to tumor cells promote tumor progression by targeting PTEN	Li et al. (2023b)
	TAMs	Ultracentrifugation	LncRNA H19	BC	—	Stabilize ULK1 promotes abnormal activation of autophagy in bladder cells	Guo et al. (2022)
	TAMs	Ultracentrifugation	lncMMPA	HCC	TAMs	Transfer lncMMPA to tumor cells and activate the glycolysis pathway to promote hepatocellular carcinoma malignancy	Xu et al. (2022b)
	NK	Exosome isolate kit	—	Leukemia; K562 cells	Tumor	Upregulate caspase three and P53 in tumor cell apoptosis signaling pathway	Mohammadi et al. (2022)
	NK	Ultracentrifugation	siRNA + Ce6	HCC; HepG2 cells	DCs, M1 macrophages, CD4^+^/CD8^+^ T cells	Directly induce tumor cell death and downregulated PD-L1	Zhang et al. (2022b)
Tumor cell or tissue		Ultracentrifugation	microRNA	NSCLC	γδ T cells	Downregulate hsa-miR-125b-5p expression, promote the secretion of IFN-γ and TNF-α, increase the activation of γδ T cells and cytotoxicity in tumor cells	Peng et al. (2020)
		No mentioned	FGF9	OC	DCs, B cells, Macrophages CD4^+^/CD8^+^ T cells, Neutrophils	Prevent malignant transformation caused by FGFR pathway and inhibit immunosuppression	Xu et al. (2022c)
		Exosome isolate kit	miR-33	BC; 4T1 cells	Macrophages	Polarize into M1-type macrophages	Moradi-Chaleshtori et al. (2021)
		Exosome isolate kit	Tumor antigens (HSP70, Her2/Neu, Mart1, TRP, and gp100)	CRC; CT26	CTLs, Treg cells	Decrease the number of Treg cells, upregulated IFN-γ, and increase CTLs infiltration	Ganji et al. (2020)
		Ultracentrifugation	PD-L1; LSD1	GC	T cells	Maintain cell membrane PD-L1 and reduce exosomal PD-L1 secretions, restores T cells’ killing function and counteracts their immunosuppressive function	Shen et al. (2022)
		Differential centrifugation	microRNA	GC; MFC cells	Macrophages, CD8^+^ T cells	Reduce PD-L1 levels in macrophages by exosomal microRNA	Wang et al. (2023a)
		Exosome isolate kit	microRNA-34a	CRC; CT26	CD8^+^ T cells	Polarization toward CD8^+^ T cell subsets, downregulate PAI-1 and VEGF-A, reduce TGF-β, IL-6, and IL-17A secretion, and promote IFN-γ secretion	Hosseini et al. (2022)
		Ultracentrifugation	—	LC; A549, LLC cells	CD8^+^ T cells, Treg cells, DCs	Trigger stronger DC-mediated immune response and reduce Treg cells in the TME	Wang et al. (2020)
		Exosome isolate kit	PTPRO	BC; MCF-7 cells	tumor-associated macrophages (TAM)	Induce macrophages convert to M1 macrophages and make STAT signals in macrophages were inactivated	Dong et al. (2021)
		No mentioned	KRT6B	BLCA; RT4, T24, BIU87, J82, UMUC3, 5637, 253J	Macrophages	M2 polarization of macrophages, regulating EMT and immune response promotes the progression of BLCA	Song et al. (2022)
		Ultracentrifugation	lncRNA SNHG16	BC	γδT cells	Induce CD73^+^γδ1 Treg cells to achieve immunosuppression	Ni et al. (2020)
		Ultracentrifugation, ExoQuick exosome precipitation solution kit	circUHRF1	HCC	NK	Induce NK cell dysfunction, degrade miR-449c5p, upregulate its expression of TIM-3 in NK cells, and cause resistance to anti-PD1 immunotherapy	Zhang et al. (2020a)
		Ultracentrifugation, ExoQuick exosome precipitation solution kit	circUSP7	NSCLC	CD8^+^ T cells	The circUSP7/miR-934/SHP2 axis induces CD8^+^ T cell dysfunction and anti-PD1 resistance	Chen et al. (2021b)
		Ultracentrifugation	—	CML/CB; K562	Treg cells	Induce T cells become tumor-favorable suppressor or regulatory T cells	Jafarzadeh et al. (2021)
		Exosome isolate kit	ER stress markers (PERK, ATF6 and GRP78); PD-L1	HNC; HN4	Macrophages	Upregulation of macrophages PD-L1 expression to drive M2 macrophage polarization	Yuan et al. (2022)
		Ultracentrifugation	miR-27a-3p	BC; MCF-7	Macrophages	Upregulate PD-L1 expression and regulate PTEN-dependent PI3K/AKT pathway promotes immune escape of breast cancer	Yao et al. (2020)
		Differential centrifugation, Iodixanol density gradient centrifugation	B7-H4	GBM	Th1 T cells	Inactivate the STAT1 pathway increases FoxP3 expression in differentiated Th1 cells and promotes immunosuppression and tumor growth	Tian et al. (2022)
		Ultracentrifugation	circGSE1	HCC	Treg cells	Regulate miR-324-5p/TGF-β1/Smad3 axis, induce Tregs expansion and promote the immune escape	Huang et al. (2022a)
		Differential centrifugation	CircRNA-safb2	RCC	M2 Macrophages	Mediate M2 macrophages polarization and promote the progression of renal cell carcinoma	Huang et al. (2022b)
		Ultracentrifugation	HRSS345D	Melanoma; B16F10 cells	CD8^+^ T cells	Drive immunosuppressive exosome secretion to induce HRS phosphorylation and limit CD8^+^ T cell infiltration into tumors	Guan et al. (2022)
		Ultracentrifugation	Lin28B	Lin28B-Hi-BC; 4TO7 cells	Neutrophils	Induce neutrophil infiltration and N2 transformation, promote immunosuppression of the pulmonary premetastatic niche and supports cancer progression	Qi et al. (2022)
		Differential centrifugation, Ultrafiltration	LncRNA KCNQ1OT1	CRC; SW1463	CD8^+^ T cells	Regulate PD-L1 ubiquitination, inhibit CD8^+^ T cell response, and mediate immune escape in colorectal cancer	Xian et al. (2021)
		Ultracentrifugation	miR-1246, p53	LC; A549	Macrophages, Th1/Th2 cells	Polarization to M2 macrophages and mitochondrial metabolism causes TME immunosuppression	Pritchard et al. (2020)
		Differential centrifugation	MicroRNA hsa-miR-31873p	Melanoma; M113, M117	CD8^+^ T cells	Downregulate TCR signaling, weaken CD8^+^ T cell immune response and drive immune escape of melanoma	Vignard et al. (2020)
		Ultracentrifugation	Dsg2	SCC	—	Downregulation of miR-146a and promotion of IL-8 secretion, influence the ability of the TME to produce larger xenograft tumors	Flemming et al. (2020)
		Ultracentrifugation	CMTM6	OSCC	M2 Macrophages	Activation of ERK1/2 signaling pathway induces polarization of M2-like macrophages	Pang et al. (2020)
		Ultrafiltration	PPARα	BC; 4T1 cells Melanoma; B16F10 cells COAD; MC38 cells	DCs	Lipids accumulate and enhance FAO activity, shift metabolism toward mitochondrial oxidative phosphorylation, and induce immunologically dysfunctional DCs that promote immune evasion	Yin et al. (2020)
		Ultracentrifugation	miR-155-5p	OC	Macrophages	Lead to reprogramming of macrophages to suppress anti-tumor immune responses	Li et al. (2022)
		Exosome isolate kit	lncARSR	RCC	Macrophages	Induce macrophage polarization through the STAT3 pathway and promote tumor progression	Zhang et al. (2022c)
		Differential centrifugation	microRNA-21	HNSCC; FaDu OECM-1 cells	CD8^+^ T cells	Promote the M2 polarization of TAMs, regulate NLRP3 phosphorylation and lysine 63-ubiquitination, inhibit the NLRP3 inflammasome activity of TAMs, and enhance cisplatin resistance	Cheng et al. (2022a)
		Exosome isolate kit	RNF126	NPC	Macrophages	Affect the immune microenvironment and promote the progression of nasopharyngeal carcinoma by regulating PTEN ubiquitination	Yu et al. (2022)
		Differential centrifugation	circVCP	CRC	TAMs	Regulate macrophage M1/M2 polarization to promote colorectal cancer progression	Tang et al. (2023b)
		Differential centrifugation	miR-183-5p	ICC	Macrophages, T cells	Activate the AKT signaling pathway, upregulate of macrophage PD-L1, inhibit T cell immunity, induce immune suppression of macrophages, and promote the progression of tumor	Luo et al. (2022)
		Differential centrifugation	miRNA	LC; HCC827	—	Regulate CD45^+^ EpCAM^+^ cells apoptosis in lung cancer	Lu et al. (2022)
		Exosome isolate kit	circRNA-ZNF451	LUAD	Macrophages: CD8^+^ T cells	Induce macrophages M2 polarization to reshape the tumor immune microenvironment and inhibit anti-PD-1 therapy	Gao et al. (2022)
		Ultracentrifugation	miR-1246	GBM	MDSC, T cells	Induce differentiation and activation of MDSCs in a DUSP3/ERK-dependent manner and facilitates the formation of an immunosuppressive microenvironment	Qiu et al. (2021)
		Ultracentrifugation	miR-142-5p	CSCC	CD8^+^ T cells	Through ARID2-DNMT1-Ifn-γ signaling to reforms lymphatic vessels and induces IDO expression, which inhibits and depletes CD8^+^ T cells	Zhou et al. (2020)

Abbreviations: Lung adenocarcinoma (LUAD), Triple-Negative Breast Cancer (TNBC), Osteosarcoma (OS), Oral squamous cells carcinoma (OSCC), Breast cancer (BC), Head and neck squamous cell carcinoma (HNSCC), Glioblastoma (GBM), Gastric carcinoma (GC), Colorectal cancer (CC), Hepatocellular carcinoma cells (HCC), Non-small cell lung carcinoma (NSCLC), Ovarian cancer (OC), Colorectal cancer (CRC), Lung cancer (LC), Head and neck cancer (HNC), Renal cell carcinoma (RCC), Squamous-cell carcinoma (SCC), Nasopharyngealcarcinoma (NPC), Intrahepatic cholangiocarcinoma (ICC), Cutaneous Squamous Cell Carcinoma (CSCC).

### 5.1 Immune activation by exosomes

Some researchers have found that exosomes derived from immune cells such as DCs, macrophages, NK, T lymphocytes, and tumor cells can promote tumor immunity**.** Exosomes secreted by DCs can activate CD4^+^ and CD8^+^ T cells because they contain MHC I, or II, HSP70, CD86, and HSP90 (Chaput et al., 2004), and are found to achieve a better antitumor immune response under IL-2 and CD80 (Wang et al., 2013). While the antitumor effect of macrophages is mainly M1-type macrophages, the exosomes secreted by them can reprogram tumor-associated macrophages (TAMs) and induce TAMs to transform into M1 macrophages with higher MHC II expression, showing enhanced phagocytosis and cross-presentation ability to slow tumor growth (Kim et al., 2023). The exosomes secreted by M1 macrophages of human peripheral blood mononuclear cells (PBMC) induced by lipopolysaccharide (LPS) and IFN-γ carrying miRNA-16-5p can specifically target and downregulate PD-L1 expression in gastric cancer cells, block the PD1/PDL1 checkpoint, reduce the immune escape of gastric cancer cells monitored by T cells, and activate T cell-dependent immune responses that inhibit tumor development *in vitro* and *in vivo* (Li et al., 2020). As an important part of innate immune responses, exosomes secreted by NK not only carry the characteristic markers of exosomes, but also the markers of NK, such as NKG2D, perforin, granzyme, and CD40L (Federici et al., 2020). Some studies have found that exosomes extracted from NK after co-culture with K562 cells can upregulate the expression of caspase three and p53 genes in tumor cells apoptotic signaling pathway to exert anti-tumor activity and exert rapid cytotoxic activity on target cells, which is more effective than intact NK (Mohammadi et al., 2022). T lymphocytes can include CD4^+^ T cells and CD8^+^ T cells because of the different CD molecules they express. Among them, CD4^+^ T cells include Th1, Th2, Th17, regulatory T (Treg) cells, and so on (Borst et al., 2018). CD8^+^ T cell can directly kill tumor cells, and the secreted exosomes also have antitumor effects. For example, in the study of melanoma cells, it has been found that administration of exosomes derived from activated CD8^+^ T cells can exhaust fibro blastoma stromal cells composed of MSCs and cancer-associated fibroblasts (CAFs), disrupt tumor progression, and result in loss of invasiveness and metastasis of melanoma cells (Seo et al., 2018). Tumor-derived exosomes can be used as carriers of tumor-related antigens to activate immune responses and have been widely studied in various tumors, such as gastric, breast, colon cancer, and melanoma. In the study of gastric cancer, tumor-derived exosomes LSD1 knockout or melatonin (MLT) treatment reduced the secretion of PD-L1 to restore T cell responses in gastric cancer (Shen et al., 2022; Wang et al., 2023). In breast cancer, exosomes containing miR-33 by targeting macrophage polarization regulatory factor MafB, downregulate M2 markers such as CD206, Arg-1, TGF-β, IL-10, and upregulate M1 markers such as CD86, NOS2, TNF-α, and IL-1β, and induced M2 to transform into M1 macrophages to inhibit tumor cell migration and invasion (Moradi-Chaleshtori et al., 2021). The exosomes from breast cancer cells overexpressing O-type protein tyrosine phosphatase receptor (PTPRO) induce the polarization of M1 macrophages by mediating the dephosphorylation of STAT3 and STAT6, which reduce migration and invasion of tumor cells (Dong et al., 2021). CT26 colorectal cancer cell-derived exosomes can carry and deliver specific tumor antigens (such as HSP70, Mart1, Her2/Neu, gp100, and TRP), reduce the number of Tregs, increase the number of CD8^+^ T cells, upregulate the level of IFN-γ, and trigger the immune response *in vivo* (Ganji et al., 2020). Exosomes are rich in miRNAs and related proteins, which are more widely used in anti-tumor research.

### 5.2 Immunosuppressive effects of exosomes

Exosomes are good for antitumor, but at the same time, in the in-depth study of tumor therapy, exosomes also inhibit or weaken the antitumor immune response, such as by mediating the TME, inhibiting immune cell function, inducing angiogenesis, tumor cell migration and proliferation, tumor immune tolerance, and immune escape. Therefore, the study of exosomes inhibiting the immune response is also very important. The inhibition of exosomes in the antitumor immune response is mainly reflected in the effect on the function of DCs, NK, and T lymphocytes. For exosomes derived from TAM, lncMMPA can be transferred to HCC cells to stabilize ALDH1A3, activate the aerobic glucose degradation pathway, lead to metabolic reprogramming of the miR548/ALDH1A3 pathway, and promote tumor proliferation (Xu et al., 2022). The exosomes derived from CAFs in TME overexpress microRNA-92, downregulate the target gene LATS2, enhance the nuclear translocation of YAP1 in the PD-L1 enhancer region, increase the transcriptional activity of PD-L1, and inhibit the function of immune cells in breast cancer through the miR-92/PD-L1 pathway (Dou et al., 2020). Studies on exosomes derived from M2 macrophages have found that they contain LncRNA H19, which can stabilize autophagy-activated kinase 1 (ULK1) and promote autophagy in bladder cancer (BLC) (Guo et al., 2022), and carry microRNA-155-5p, which can promote colon cancer immune escape through down-regulating ZC3H12B (Ma et al., 2021), and carry miRNA-23a-3p, which promotes oral squamous cell carcinoma (OSCC) progression by targeting PTEN (Li et al., 2023). By reducing the activity of CD8^+^ T cell and MHC I expression in DCs, exosomes secreted by CD8^+^ T cells can inhibit antitumor effects. (Xie et al., 2010). For tumor cell-derived exosomes, their own tumor-derived receptors, proteins, and RNA can be transferred to the recipient cells, interfering with the immune activity of APCs and the antigen presentation process, which is beneficial to the TME. At the same time, tumor-derived exosomes also have immunosuppressive molecules on the surface or secrete immunosuppressive factors, which can negatively regulate the innate and adaptive immune responses of tumors. For example, HSP72 and HSP105 on the surface of tumor exosomes can promote tumor metastasis by inducing IL-4 secretion from DCs in a TLR6 and TLR2-dependent manner. Exosomes released from lung and breast cancers can inhibit maturation and migration of DCs and promote immunosuppression (Ning et al., 2018; Yin et al., 2020). The lung cancer cell-derived exosomes can also induce M2 macrophage polarization (Pritchard et al., 2020), and regulate CD45^+^EpCAM^+^ cells apoptosis in lung cancer (Lu et al., 2022), and carry circUSP7, which can regulate the miR-934/SHP2 axis in small cell lung cancer (NSCLC) to induce CD8^+^ T cell dysfunction and anti-PD-1 resistance (Chen et al., 2021). Breast cancer cell-derived exosomes transmit the lncRNA SNHG16 to induce CD73^+^γδ1 Treg cells (Ni et al., 2020), miR-27a-3p on exosomes induced by endoplasmic reticulum stress can regulate PD-L1 expression in macrophages to promote immune escape of breast cancer (Yao et al., 2020), and Lin28B-hyper-expressed exosomes can promote immunosuppression and cancer progression in the premetastatic niche in the lung (Qi et al., 2022). The HCC-derived exosomes with highly express circUHRF1 and circGSE1, which can not only inhibit the secretion of NK cell-derived IFN-γ and TNF-α, degrade miR-449c-5p, and upregulate TIM-3 expression, so as to reduce the number of NK and tumor invasion, induce NK function exhaustion (Zhang et al., 2020), and also regulate the miR-324-5p/TGFBR1/Smad3 axis to induce Tregs expansion, inhibit CD8^+^ T cells functions, and cause tumor immune escape (Huang et al., 2022). The immunosuppressive effects of exosomes also include inducing EMT and regulating the immune microenvironment to promote invasion and metastasis (Song et al., 2022), increasing FoxP3 expression of differentiated Th1 cells (Tian et al., 2022), driving differentiation and activation of Bone marrow-derived suppressor cells (Qiu et al., 2021), remodeling lymphatic vessels and inducing IDO to promote the TME (Zhou et al., 2020), and regulating PD-L1 ubiquitination to mediate immune escape through MiR-30a-5p/USP22 (Xian et al., 2021). As the role of exosomes in tumor immune regulation is gradually revealed, researchers have a deeper understanding of exosomes. Studies on how exosomes participate in anti-tumor immunosuppression can be better used in tumors therapy.

## 6 Exosomes as delivery carriers in tumor immunity

**FIGURE 5:**
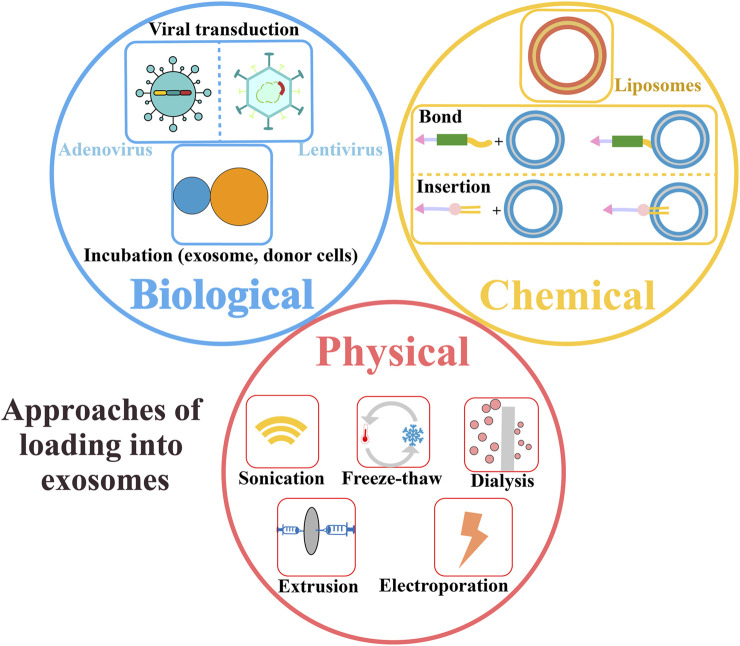
Approaches of loading exosomes as carriers, including physical, chemical, and biological methods.

The research on drug delivery systems is a hot topic in the field of biomedicine, and the traditional delivery carriers include lipid nanoparticles, liposomes, virus, polymeric and inorganic nanoparticles (Yousefpour and Chilkoti, 2014). As a new generation of natural biological nanoscale carriers, exosomes have attracted the attention of scholars in the field of tumor therapy. The exosomes have the same characteristics as the cell membrane of donor cells, such as negative charge, reduce clearance of MPS, prolong circulation time and immune escape. In addition, they have the advantages of high biocompatibility, stability, homing ability, low toxicity, immunogenicity, and crossing the blood-brain barrier and other biological barriers. They are often used as a natural endogenous carrier. Exosomes express specific molecules (such as tetraspanins and integrins) on their surface, which can fuse with specific cells or organs, making them natural targeting (Vader et al., 2016; Yang et al., 2018). When exosomes are engineered or surface modified, they can optimize their natural targeting, reduce the damage to normal tissues or cells, and reduce the toxicity or side effects of their drugs (Mehryab et al., 2020; Zhan et al., 2020). For targeted therapy, exosomes can increase drug concentration and accumulation at tumor sites, improve drug absorption, prolong drug release time and duration, reduce the accumulation in metabolic organs such as liver and kidney, prolong internal circulation time, or convert light energy into heat energy to improve tumor killing effect (Zhao et al., 2020). For example, exosomes expressing integrin α3β1 can specifically target endocyclic nonapeptide-LXY30 to reduce the uptake of parental cells (Carney et al., 2017). Exosomes expressing integrin αvβ5, α6β4 and α6β1 can specifically bind to Kupffer cells, fibroblasts and epithelial cells to promote tumor metastasis (Myint et al., 2020). For example, tetraspanin CD47 can bind to signal regulatory protein α (SIRPα) and send a signal of “do not eat me” to avoid phagocytosis (Logtenberg et al., 2020). At present, to optimize the targeting of exosomes and improve the tumor killing effect, molecular cloning, lentivirus packaging technology, bioorthogonal chemistry, etc., can be used for targeted peptide or receptor protein engineering modification (He et al., 2022), magnetic nanoparticles, targeted drugs and other physical modifications (Ferreira et al., 2022), covalent modification, click chemistry for surface chemical modification (Xu et al., 2020). Ligand/receptor interaction is chemically modified (He et al., 2022). And these advantages solve the safety and delivery efficiency problems faced by other delivery carriers. When combined with other therapies, tumor growth can be inhibited more effectively. Therefore, exosomes have great potential to be delivery carriers for the delivery of various small molecules and biological macromolecules (including proteins and peptides, nucleic acids, and genes) (Table 2). At present, exosomes can be loaded in passive ways before isolation (indirect loading) and active ways after isolation (direct loading), including passive ways such as engineering modification before isolation (such as transfection) and co-culture of drugs with exosome-derived cells, and active ways such as sonication, freeze-thaw cycles, extrusion, incubation, and electroporation after separation (Figure 5).

**TABLE 2:** Tumor therapy with exosomes as delivery carriers.

Exosome origin		Isolation	Loading cargos	Loading method	Tumor type	Administration route	Study outcome	Refs
Normal cell or tissue	Normal cell	Ultracentrifugation	GMP-AMP	Incubation	Melanoma; B16F10 cells	Intratumor injection	Inhibit tumor growth, activate the STINGa pathway, and enhances antitumor innate and adaptive immune responses	McAndrews et al. (2021)
	Normal cell	Ultracentrifugation	cGAMP	Incubation	Melanoma; B16F10 cells	Intratumor injection	Improve combined cancer immunotherapy for tumor suppression, activation of immune responses and suppression of immune escape	Fan et al. (2021)
	Normal cell	Ultracentrifugation	Ce6, R848	Incubation	PCC; RM-1 cells	Intratumor injection	Sonodynamic therapy combined with immunotherapy, induction of DC, M1-like phenotype reprogramming, activate effector T cells, reversal of the immunosuppressive tumor microenvironment	Wang et al. (2022a)
	Normal cell	Sucrose density gradient centrifugation	PH20, FA	Transfection	BC; 4T1 cells	Intravenous injection	Dual targeting enhances the anti-tumor effect of chemotherapy, reverse immunosuppression, regulate the TME to improve therapeutic efficiency, and reduce tumor cell metastasis	Feng et al. (2021)
	Normal cell	Ultracentrifugation	aCD3-aHER2	Transfection	BC; HCC 1954	Intravenous injection	Redirection and activation of cytotoxic T cells, dual targeting of T cell CD3 and breast cancer-associated HER2 receptor, show efficient and specific tumor-targeted immunotherapy	Shi et al. (2020)
	Normal cell	Density gradient centrifugation	T7 peptide	Transfection	GBM; GL261 cells	—	Stimulate of tumor macrophages repolarization to M1-type repolarization, enhance their phagocytosis and CD8^+^ T cells invasion, and limit the immunosuppression of GBM	Li et al. (2023a)
	BM-MSC	Differential centrifugation, Sucrose density gradient centrifugation	Oxaliplatin prodrug (OXA-MAL)	Vortex Incubation	PDAC; PANC-02 cells	Intravenous injection	Enhance ICD induction, improve DC maturation, reverse immunosuppression, and increase infiltration of antitumor CLTs induce effective innate and adaptive immunity	Zhou et al. (2021b)
	NSC	Exosome isolate kit	Antisense oligonucleotides (ASO)	Incubation with donor cells	GBM; U251 cells	Intratumor injection	Effectively stimulate the immune activity of DCs or mouse macrophages for achieving antitumor effect	Adamus et al. (2022)
	Tissue fluid	Sonication	Tanghinin IIA (TanIIA) and Glycyrrhizic acid (GL) Self-assembled nanomicelle (TGM)	Sonication	GBM; GL261 cells	Intravenous injection	Release of chemotherapeutic drugs across the blood-brain barrier, promote the maturation of DCs, induce M1 macrophages polarization and enhance the antitumor efficacy by the combination chemoimmunotherapy	Cui et al. (2023)
	Tissue fluid	Exosome isolate kit	DOX-RB	Incubation, Sonication	Melanoma; B16F10 cells	Intravenous injection	Improve targeting, induce extracellular leakage, and the application of alternating magnetic fields can activate thermochemtherapy of deep tumors and enhance T-cell infiltration at pulmonary metastases	Shen et al. (2020)
	Milk	Ultracentrifugation	DOX- endoperoxide-Ce6) (EPT1)	C=N Conjugation	OSCC; HSC-3, SCC-9, CAL-27 cells	Intravenous injection	Construction of a pH/light-sensitive drug system, combine chemotherapy and singlet oxygen released by thermal cyclization to enhance antitumor activity	Zhang et al. (2020b)
Immune cell	CAR-T	Ultracentrifugation	Mesothelin (MSLN)	Transfection	TNBC; BT-549, MSLN MDA-MB-231, B-NDG cells	Intravenous injection	Exosomes carry perforin and granzyme B, and dual target to inhibit tumor growth	Yang et al. (2021)
	DCs	Ultracentrifugation	Fluorouracil (FU)	Electroporation	CRC; CT26 cells	Intravenous injection	Combine chemotherapy can inhibit tumor cell proliferation and induce apoptosis, and enhance antitumor efficacy	Xu et al. (2020b)
	DCs	Ultracentrifugation	CD62L-OX40L	Transfection	BC; 4T1 cells	Intravenous injection	Activation of effector T cells and inhibition of Treg induction, restore the immunosuppressive microenvironment, induce immune amplification, and cooperate with lymph node homing to enhance the immunotherapy of metastatic breast cancer	Ji et al. (2021)
	Macrophages	Ultracentrifugation	Docetaxel (DTX)	Electroporation	BC; 4T1 cells	Intravenous injection	Combine chemotherapy and immunotherapy reactivate the tumor immune microenvironment and improve the antitumor efficacy	Zhao et al. (2022)
	Macrophages	Differential speed centrifugation	Thalidomide (THD)	Extrusion	No mentioned	—	Regulate Treg cell proliferation and inhibit tumor growth in a dose-dependent manner	Yang et al. (2023)
	Macrophages	Ultracentrifugation	Paclitaxel	Sonication	BC; 4T1 cells	Intravenous injection	Activate macrophage-mediated inflammation to enhance paclitaxel antitumor activity	Wang et al. (2019a)
	Macrophages	Ultracentrifugation	Functional DNA QDs	Biocompatible interconnects	BC; 4T1 cells	Intravenous injection	Construct target-triggered drug delivery systems for tumor imaging and therapy	Fan et al. (2019)
	Neutrophils	Ultracentrifugation, Density gradient centrifugation	SPIONs, DOX	Extrusion	GC; HGC27 cells	Intravenous injection	Activate apoptosis signaling pathway, dual-targeting, and chemotherapy immune combination antitumor	Zhang et al. (2022a)
	NK	Differential speed centrifugation	Cis platinum	Electroporation	OC; SKOV3, COC1/DDP, ISOE80 cells	—	Enhance cytotoxicity and reactivates NK cells, functions enhanced antitumor effects in ovarian cancer	Luo et al. (2023)
	NK	Exo-spin™ kit	Sorafenib (SFB)	Electroporation	TNBC; MDA-MB-231 cells	—	Improve cytotoxicity, targeted inhibition of tumor cells proliferation and enhance apoptosis	Hashemi et al. (2023)
	NK	Ultrafiltration, Size exclusion chromatography	microRNA (miR)-186	Transfection	GBM; CHLA-136 cells	Intravenous injection	Inhibit of neuroblastoma tumorigenesis and block TGFβ1-dependent NK cell suppression/immune escape	Neviani et al. (2019)
	Macrophages, Tumor, Milk	EV-precipitation	Zinc phthalocyanine (ZnPc)	Incubation	CRC; MC38 cells	Intravenous injection	Enhance photodynamic therapy, induce ICD, promote DCs maturation, and induce immune memory for antitumor	Huis et al. (2022)
Tumor cell or tissue		Ultracentrifugation	AuNRs	Incubation	HCC, HepG2 cells BC, MCF-7 cells CRC, HCT116 cells LC, A549 cells	—	Combine photothermal therapy induces apoptosis and enhance antitumor efficacy	Zheng et al. (2020)
		Exosome isolate kit	POM1, Metformin	Electroporation	Melanoma; B16F10 cells	Intravenous injection	Combination with immunometabolism therapy, reshape energy metabolism to activate the innate and adaptive immune systems	Wu et al. (2022a)
		Ultracentrifugation	Ce6	Sonication	Melanoma; B16F10 cells	Intravenous injection	Photoacoustic imaging-guided photodynamic therapy and immunotherapy combined with antitumor therapy	Jang et al. (2021)
		Ultracentrifugation	Thermosensitive liposomes of ICG/R837	Freeze-thaw	CRC; CT26 cells	Intravenous injection	Generate tumor-associated antigens, promotes DCs maturation under R837, enhances CD8^+^ and CD4^+^ T cells infiltration in tumor	Cheng et al. (2021)
		Exosome isolate kit	CD47 inhibitor, Cisplatin (CDDP)	ExoFectin sRNA-into-Exosome kit	LC; A549, LLC cells	Intravenous injection	CD47 antagonist can improve the resistance to cisplatin by changing the immune microenvironment, increase T cells proliferation, and enhance the targeted antitumor effect	Cui et al. (2022)
		Ultracentrifugation	TLR9 ligand K-type CpG ODN, TLR3 ligand p (I:C)	Freeze dried Hydration	BC; 4T1 cells	Intraperitoneal injection	Formation of Th1 and proinflammatory cytokines, activate CD4^+^ and CD8^+^ T cell responses, initiate humoral and cell-mediated tumor-specific immune responses, overcome the Treg cell functions immunosuppressive in the TME	Yildirim et al. (2021)
		Ultracentrifugation	Temozolomide (TMZ), Dihydrotanshinone (DHT)	Sonication	GBM; GL261 cells	Intravenous injection	Upregulation of caspase-3 promotes tumor cell apoptosis, downregulation of MGMT and P-gp expression reduces drug resistance, enhance antitumor activity, and trigger an immune response	Wang et al. (2022b)
		EV-precipitation	Zinc phthalocyanine (ZnPc)	Incubation, Size exclusion chromatography	CRC; MC38, B16F10 cells	Intravenous injection, Intratumor injection	Selectively targeting tumors to improve photodynamic therapy and enhance the antitumor effect	Lara et al. (2021)
		Differential centrifugation, Sucrose density gradient centrifugation	CCL22 siRNA	Electroporation	PDAC; PANC-02 cells	Intramuscular injection	Block the CCR4/CCL22 axis between DCs and Treg cells, and inhibit Treg cell expansion, can be used as an effective preventive vaccine to delay tumor growth	Zhou et al. (2022)
		Exosome isolate kit	miRNA-155	Electroporation	—	—	By delivering miRNA and inducing DC maturation, the proliferative capacity of T lymphocytes was significantly increased	Asadirad et al. (2019)
		Ultracentrifugation, Sucrose density gradient centrifugation	IFN-γ/Calpain inhibitor III	Incubation with donor cells	LC; LLC-1 cells	Intravenous injection	Targeting ORAI1 calcium channel can increase immune activation response, inhibit tumor growth in an immune-dependent manner, and promote systemic anti-tumor immunity	Chen et al. (2022b)
		Ultracentrifugation	Lipids	Sonication	BC; 4T1 cells	Intravenous injection	Inhibition of Kupffer cell-mediated phagocytosis and ability to effectively target tumor metastasis and promote lung distribution of therapeutic nanocarriers	Qiu et al. (2019)
		Ultracentrifugation	YTHDF1	Exosome transfection kit	Melanoma; B16F10 cells	Intratumor injection	Targeting YTHDF1 causes YTHDF1 depletion, promotes MHC-I degradation, drives immune escape and immune checkpoint inhibitors resistance, restores tumor immune surveillance	Lin et al. (2023)

Abbreviations: Prostatic cancer (PCa), Breast cancer (BC), Glioblastoma (GBM), Pancreatic ductal adenocarcinoma (PDAC), Oral squamous cell carcinoma (OSCC), Triple-Negative Breast Cancer (TNBC), Colorectal cancer (CRC), Gastric carcinoma (GC), Ovarian cancer (OC), Hepatocellular carcinoma cells (HCC), Lung cancer (LC).

### 6.1 Application of exosomes as carriers for loading small molecules

Many studies have found that exosomes can increase the accumulation of drugs in target cells, improve the stability of small molecular drugs and blood circulation time, to improve the therapeutic effect of small molecular drugs. Exosomes can load small molecules chemotherapy drugs, photosensitizers, photothermal agents, tumor immune-related metabolic agonists, and other drug molecules for tumor therapy.

#### 6.1.1 Chemotherapy small molecule drugs

It has been reported that exosomes can be loaded with small molecular drugs related to chemotherapy. For example, exosomes derived from DCs loaded with fluorouracil (FU) by electroporation can effectively inhibit tumor cell migration, proliferation, and apoptosis, enhancing their the effectiveness against colon cancer (Xu et al., 2020). As another example, M1 macrophage-derived exosomes loaded with PTX can polarize macrophages and release pro-inflammatory factors, which can not only participate in macrophage programming targeting mitochondrial functions and reactivate the tumor immune microenvironment, but also enhance antitumor effects through caspase-3-mediated pathways, thus treating breast cancer (Wang et al., 2019; Zhao et al., 2022). However, exosomes derived from RAW264.7 cells and thalidomide (THD)-liposome co-ultrasonic extrusion can eliminate the expansion and proliferation of Treg cells induced by TNF *in vitro*, and reduce the survival and infiltration of Treg cells in the TME *in vivo*, so as to inhibit tumor growth (Yang et al., 2023). Exosomes derived from NK cells, have typical protein markers of NK cell, and have anti-tumor effects. They are used to deliver cisplatin to enhance cytotoxic effects on ovarian cancer (OC), reactivate NK cell function, and reverse immunosuppression, thus enhance the therapeutic effect of anticancer drugs (Luo et al., 2023), used to deliver sorafenib (SFB), which can enhance tumor cytotoxicity, inhibit proliferation and induce apoptosis in triple-negative breast cancer cells (Hashemi et al., 2023). Superparamagnetic iron oxide nanoparticles (SPION) are used to modify exosomes derived from neutrophils in peripheral blood from the body and DOX, and the apoptosis pathway can be activated after extrusion of the two to achieve dual-targeting characteristics and a combined antitumor response (Zhang et al., 2022). Exosomes and DOX-iron oxide can also be used to form nano-raspberry, which can be used as a targeting agent for tumor metastasis and T cell infiltration for the treatment of deep tumors (Shen et al., 2020). In addition, DOX can also be loaded with exosomes derived from tumor cells, which has good tumor targeting, such as liver cancer, breast cancer and melanoma. It can show good tumor inhibition and killing ability, which improves a new idea for the application of exosome biomimetic nano-drugs derived from tumor cells in tumor therapy (Yong et al., 2019).

#### 6.1.2 Phototherapy materials and other drugs

Exosomes can be used in cooperation with phototherapy for antitumor purposes. For example, milk-derived exosomes and DOX are connected by an imine bond and then loaded with endoperoxide derivative (EPT1) and dihydroporphyrin e6 (Ce6), which can not only release DOX specifically at the tumor site through pH, but also accelerate the production of singlet oxygen ROS EPT1 under NIR irradiation, thus achieving photochemical synergistic therapy for OSCC, which opened up a new therapeutic idea of using milk exosomes as a potential stable delivery carrier. (Zhang et al., 2020). Ce6 can also be loaded into exosomes derived from pancreatic cancer MIA-PaCa-2 cells, promote immune cell proliferation and secretion of cytokines, and generate reactive oxygen species inside B_16_F_10_ cells by laser irradiation (Jang et al., 2021). Photothermal therapy is a type of phototherapy, and the carriers mostly use exosome derived from tumor cells. Researchers load gold nanorods (AuNRs) with exosomes derived from aptamer-modified HepG2 cells, which had better specificity and photothermal killing of tumors (Zheng et al., 2020). The exosomes secreted by CT26 cells are loaded with ICG and R837, and under laser irradiation, the tumor-associated antigens of immunogenic cell death (ICD) are generated, the maturation of DCs is promoted under R837, and the tumor invasion of CD8^+^ T cells and CD4^+^ T cells is enhanced, so as to realize the antitumor effect of PTT (photothermal therapy) combined with immunotherapy (Cheng et al., 2021). Some studies have shown that affecting immune metabolism can also be used as an antitumor agent. Loading the extracellular enzyme CD39 antagonist POM1 and the AMP-activated protein kinase agonist metformin into exosomes secreted by B_16_F_10_ cell by electroporation, which can increase extracellular ATP, promote metabolic reprogramming of TME, and activate the innate and adaptive immune systems, thereby generating synergistic anti-tumor immune responses (Wu et al., 2022). Compared with traditional synthetic drug delivery systems, many studies have shown that exosome-loaded drugs have natural advantages of lower side effects, higher efficacy, and high bioavailability.

### 6.2 Application of exosomes as carriers for loading biological macromolecules

Exosomes are naturally derived lipid vesicles, which have the advantages of high biocompatibility, low immunogenicity, and homing. They can carry endogenous drugs and protect drug activity and stability. Therefore, exosomes have become a new carrier for macromolecular drug delivery of proteins, peptides, nucleic acids, and genes and have the prospect of treating a variety of tumors.

#### 6.2.1 Protein drugs

Exosomes can be loaded with endogenous proteins by engineering means direct transfection, co-incubation (such as lentivirus or plasmid transfection) for synergistic immunotherapy of tumors. For example, Expi293F cells can be engineered to express CD3 and EGFR antibodies and PD-1 and OX40L ligands and secrete exosomes expressing antibodies, immune checkpoints, and ligands. This multi-functionalization allows CTL cells to relocate to EGFR-positive triple-negative breast cancer (TNBC) tumors while blocking the PD-L1/L2 immunosuppressive pathway on the tumor surface and activating the immune checkpoint stimulation signal (Cheng et al., 2022). In addition, Expi293F cells can also be designed to express CD3 and HER2 antibodies to secrete and express multivalent antibodies to redirect exosomes, dual target T cell CD3 and breast cancer-related HER2 receptors, and activate CTL cells to target HER2-positive breast cancer cells (Shi et al., 2020). Similarly, 293T cells can be designed to express PH20, secrete exosomes expressing PH20, and then be modified with folic acid (FA), which can regulate the TME, reverse immunosuppression, enhance cell targeting, and inhibit tumor cell metastasis (Feng et al., 2021). Exosomes derived from CD62L and OX40L-expressing DC cells have the ability to lymph node homing and immune amplification, thus enhancing the immunotherapy effect of TNBC (Ji et al., 2021). Exosomes expressed by MSLN-expressing CAR-T cells secrete perforin and granzyme B and target MSLN-positive TNBC cells (Yang et al., 2021). Researchers expressed CD47 on CT26 cells and made the secreted exosomes carry CD47 to block the interaction between CD47 and SIRPα to promote CT26 cells phagocytosis mediated by M1 macrophages and prolong the blood circulation time *in vivo* (Cheng et al., 2021). T7 peptide is modified on 293T cells, and its exosomes are used to deliver Galectin-9 siRNA, which can promote macrophage repolarization and inhibit the immunosuppression of glioblastoma (GBM) (Li et al., 2023). These results show that exosome is an effective carrier for protein drug delivery.

#### 6.2.2 Nucleic acid and gene drugs

Nucleic acid and gene drugs are a kind of biological macromolecular substances, which are difficult to deliver effectively *in vivo*. Exosomes can load nucleic acids and genes drugs to regulate tumor immunity. For example, neural stem cell-derived exosomes are loaded with Cpg-binding antisense oligonucleotides (ASO), which, on the one hand, allow tumor homing to be transferred to the glioma microenvironment and on the other hand, activate DCs and macrophages and break tolerance by immune stimulation (Adamus et al., 2022). Another example is the design of functionalized DNA as quantum dots (QD), which can be expressed on macrophage-derived exosomes for tumor imaging and therapy (Fan et al., 2019). The main gene drugs loaded by exosomes are miRNA, siRNA, and mRNA. It has been reported that miRNA-155 can promote DCs maturation and load it into exosomes derived from CT26 cells. The *in vitro* experiments showed that T lymphocytes proliferation ability in DCs treated with exosomes loaded with miRNA-155 are significantly increased, and the maturation level of DCs induced by miRNA-155 is not significantly different from that of LPS (Asadirad et al., 2019). For miRNA-186 and miRNA-34a, loading them into exosomes can inhibit tumor cell immune escape (Neviani et al., 2019; Hosseini et al., 2022). Electroporation of CCL22 siRNA into exosomes derived from PANC-02 cells induced by mitoxantrone (MTX), which can block the CCR4/CCL22 axis between DCs and Treg cells to inhibit the expansion of Treg cells, and intramuscular injection of exosomes containing CCL22 can delay the growth of pancreatic cancer, so it is expected to become an effective preventive vaccine (Zhou et al., 2022). It has been reported in the literature that exosomes loaded with biological macromolecules can retain the original activity of biomolecules, reduce immunogenicity and toxicity, ensure specific targeting (Yuan and Li, 2019), regulate the immune system at the molecular level, and inhibit tumor growth.

## 7 Limitation

It has to admit there are some limitations of the review. It is known that the field of exosomes is relatively emerging and developing fast, and is currently connected to interdisciplinary areas. Thus, it may lead to some incomplete contents covered, although we have tried our best to make comprehensive review on the progresses of the applications of exosomes in tumor immunity. Some new discoveries on exosome-related phenomena were reported without clear mechanisms interpreted, and thus some of the studies described in this review may not be completely explained in mechanisms, which need to be further explored. Furthermore, with the rapid development of technologies, the isolation and characterization methods of exosomes are also gradually increasing and updating. This review attempts to summarize the general methods and techniques and introduces some new strategies, which may not fully cover all the knowledges, considering the limited space and the focus of the work. Currently, standards on exosome research are still being developed and updated at times. Regarding the sources of exosomes, they can be from a variety of biological fluids, and it is difficult to identify the parent cell of exosomes *in vivo*. Exosome biogenesis involves the intersection of a variety of complex mechanisms, and exosomes carry a variety of molecules, such as proteins, nucleic acids, and lipids. The dominant mechanism of the multiple effects of exosomes and the contribution of multiple components are difficult to identify. In the cases of targeting, some of the intracellular mechanisms, gene expressions and physiological effects of exosomes on recipient cells are not fully clear. The above issues sometimes prevent an exosome-related review from perfect arguments. However, we still think routine update and summary of the field is significant and hope it will inspire some new ideas for the researchers.

## 8 Conclusion and prospects

As a research hotspot in recent years, exosomes have aroused as one of the great interests among the researchers. The exosomes are mediator for the exchange of information and materials between cells and participate in various important pathophysiological processes in the body. As researchers pay more and more attention to exosome function, significant advances have been made in the understanding of exosome biology and its application in the field of tumor immunotherapy. Firstly, we have a better understanding of the fundamental properties of exosomes (such as biogenesis, composition, and so on). Secondly, significant progress has been made in the application of exosomes, such as regulating tumor immune responses and delivering anticancer drugs or biomolecule drugs. Thirdly, the researchers also studied ways to improve the stability and therapeutic effectiveness of exosomes and expand therapeutic uses. This article reviews the research progress of exosomes as immunomodulatory molecules and drug delivery carriers and reveals the potential applications of exosomes in tumor immunity, including the dual functions of inhibiting tumor and promoting tumor growth, and use as a delivery carrier for antitumor immunotherapy.

**FIGURE 6:**
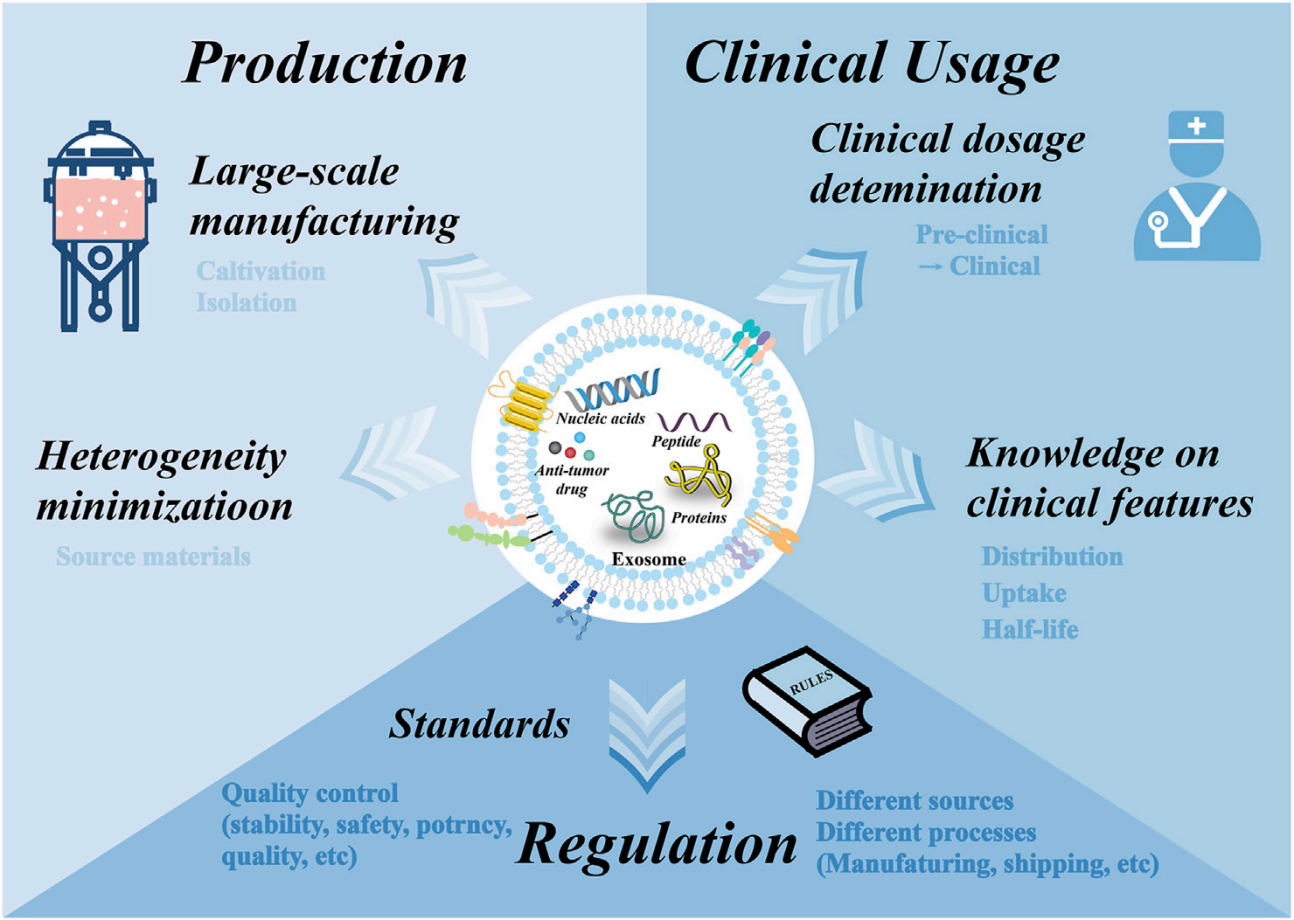
Challenges limiting the use of exosomes in tumor therapy.

However, as an emerging field, the application of exosomes in tumor immunity still faces serious challenges, such as understanding of structural composition and biological mechanisms, safety and clinical translation, industrialization of production and so on (Figure 6). First, it is necessary to improve technology to determine the mechanism of exosome release, uptake, and specific molecular targeting, to deeper understanding of exosomal functional and structural characteristics from different sources, to understand the gene expression affected by exosomes, and to understand the actual role of exosomes in the occurrence, development, and treatment of tumors. Tumor immune regulation mediated by exosomes is mainly the functional interaction between immune cells and tumor cells, and its therapeutic application in antitumor immunity may be a new option for tumor treatment, but its application in tumors is still not fully explored. Therefore, the understanding of exosomes will help to decipher the mechanism of tumor invasiveness, lay the foundation for the development of potential new exosome-based therapeutic methods, and promote the development of drugs for tumor immunotherapy. While the size, surface antigen and components of exosomes derived from different cell sources or from the same parental cells may be different (Soekmadji et al., 2018), and the mechanism of their various effects needs to be explored. Improving gene editing, labeling RNA and other technologies (Soekmadji et al., 2018), and exploring the cargo sorting mechanism to determine whether the effect of exosomes is related to the carrying of specific molecules. So, it is necessary to discover the key components of exosome therapy that cause therapeutic effects or side effects, to determine the dose, durability and experimental “control” of exosome therapy (Tzng et al., 2023), and to optimize engineering and physicochemical techniques (e.g., molecular methods such as gene overexpression and mechanical methods such as supramagnetic paramagnetic nanoparticles) to improve the effective tumor targeting of exosomes (Mardi et al., 2023), further clinical researches are needed to verify and promote the clinical transformation of exosomes. Then the development of new technologies (gene co-expression, overexpression of mRNA) or optimization technology (such as magnetization of metal oxide, membrane separation, microfluidic, immunoaffinity chromatography) (Xiang et al., 2021; Chen et al., 2022) and further basic research, to produce exosomes with controllable quality on a large scale and promote the industrialization of exosome production (scale, purity, consistency, and standardization), improving the short half-life of exosome, zeta potential difference and other internal problems, and continue to expand the application of exosomes (Chen et al., 2022). Finally, it is necessary to optimize exosome-standardized cargo loading strategies (such as ultrasound, pH gradient, and hybridization and fusion with liposomes), as well as the toxicity, stability, and biological efficacy of exosomes after they are processed and modified or loaded with drugs, especially in the field of tumor applications (Hussen et al., 2022). In conclusion, due to the unique biological properties of exosomes, exosomes may be a potential tool for tumor therapy. Further research and modification of exosomes will help to solve the above problems and promote the clinical application of exosomes as immunomodulatory molecules and drug delivery carriers.
